# Anterior shoulder dislocation and concomitant fracture of the greater tuberosity: Clinical and radiological results

**DOI:** 10.1007/s11678-018-0451-7

**Published:** 2018-03-12

**Authors:** Florian Dussing, Fabian Plachel, Teresa Grossauer, Thomas Hoffelner, Eva Schulz, Arvind von Keudell, Alexander Auffarth, Philipp Moroder

**Affiliations:** 1 Department of Traumatology and Sports Injuries, Paracelsus Medical University, Müllner Hauptstraße 48, 5020 Salzburg, Austria; 2 Department for Shoulder and Elbow Surgery, Center for Musculoskeletal Surgery, Charitè – Universitätsmedizin Berlin, Berlin, Germany; 3 Department of Orthopaedic Surgery, Brigham and Women’s Hospital, Harvard Medical School, Boston, USA

**Keywords:** Shoulder fractures, Conservative treatment, Surgery, Recurrence, Range of motion, Schulterfrakturen, Konservative Behandlung, Operation, Rezidiv, Bewegungsumfang

## Abstract

**Background:**

Recurrence rates after primary traumatic shoulder dislocation are distinctly high. We hypothesized that concomitant isolated fractures of the greater tuberosity are associated with low rates of persistent instability but decreased range of motion.

**Methods:**

Between 2007 and 2013, 66 consecutive shoulders in 64 patients were treated for primary shoulder dislocation combined with an isolated fracture of the greater tuberosity with either a nonsurgical (48 shoulders, 72.7%) or surgical (18 shoulders, 27.3%) treatment approach. In all, 55 cases (83.3%) were available for clinical follow-up examination after an average of 59.0 ± 20.7 months (range: 25–96 months) and of these, 48 (72.7%) patients consented to radiological evaluation to determine healing and position of the greater tuberosity.

**Results:**

The mean range of motion of the affected shoulder was significantly decreased by 9° of elevation (*p* = 0.016), 11° of abduction (*p* = 0.048), 9° of external rotation in 0° of abduction (*p* = 0.005), and 10° of external rotation in 90° of abduction (*p* = 0.001), compared with the unaffected shoulder. The mean WOSI score was 373 ± 486 points, the mean Constant and Murley score was 75.1 ± 19.4 points, and the mean Rowe score was 83 ± 20 points. Three cases (5.5%) of re-dislocation were reported among the cohort, all of them were due to a relevant trauma. Radiological evaluation revealed anatomically healed fragments in 31 shoulders (65%), dislocation of the fragment in ten shoulders (21%), impaction into the humeral head in four shoulders (8%), and absorption in three shoulders (6%).

**Conclusion:**

A concomitant isolated fracture of the greater tuberosity leads to low recurrence rates along with a significant decrease in range of motion after primary traumatic anterior shoulder dislocation.

## Zusammenfassung

**Hintergrund:**

Die Reluxationsrate nach erstmaliger traumatischer vorderer Schulterluxation ist generell hoch. Eine begleitende Fraktur des Tuberculum majus wird als protektiver Faktor angesehen, könnte jedoch einen negativen Einfluss auf den Bewegungsumfang haben.

**Methodik:**

Zwischen 2007 und 2013 wurden 66 Schulterverletzungen mit dem genannten Verletzungsbild entweder nach konservativem (48 Schultern, 72,7 %) oder chirurgischem (18 Schultern, 27,3 %) Behandlungsansatz an der Abteilung der Autoren versorgt. Davon wurden 55 Schultern (83,3 %) klinisch und 48 (72,7 %) auch radiologisch nachuntersucht. Mittels detaillierter Anamnese, 4 klinischen Scores – Constant-Murley Score, WOSI (Western Ontario Shoulder Instability Index), Rowe Score, subjektiver Schulterwert („subjective shoulder value“, SVV) – und Bestimmung des Bewegungsumfangs im Schultergelenk wurden Schulterfunktion und Schulterstabilität erhoben. Eine etwaige Dislokation des Tuberculum majus wurde mittels Röntgenaufnahmen in 3 Ebenen (a.-p., seitlich/Y-View, axial) analysiert.

**Ergebnis:**

Der Bewegungsumfang der verletzten Schulter war im Vergleich zur Gegenseite durchschnittlich um 9° (*p* = 0,016) in der Elevation, um 11° (*p* = 0,048) in der Abduktion, um 9° (*p* = 0,005) in der Außenrotation aus Neutralstellung und um 10° (*p* = 0,001) in der Außenrotation aus 90° Abduktion signifikant eingeschränkt. Der durchschnittliche WOSI-Score betrug 373 ± 486 Punkte, der Constant-Murley Score betrug 75 ± 19 Punkte und der durchschnittliche Rowe Score betrug 83 ± 20 Punkte. In 3 Fällen (5,5 %) trat eine erneute Schulterluxation in der Kohorte auf, wobei sich 2 Fälle bilateral bei einem jugendlichen Epileptiker ereigneten. Die radiologische Auswertung ergab 31 (65 %) anatomisch eingeheilte Tuberkula, 10 Fälle (21 %), in denen das Fragment disloziert war, 4 Fälle (8 %), in denen das Tuberkulum in den Humerus impaktiert war, und 3 Fälle (6 %), in denen es zur Absorption des Bruchstücks gekommen war.

**Schlussfolgerung:**

Eine begleitende isolierte Fraktur des Tuberculum majus verringert das Reluxationsrisiko nach vorderer Schulterluxation, führt jedoch gleichzeitig zu einer signifikanten Abnahme des Bewegungsumfangs.

## Introduction

The incidence of traumatic shoulder dislocations has been reported to range from 17 to 23.9/100,000 [1–4]. Recurrence rates after primary dislocation are known to be high and have been shown to depend especially on the patient’s age, as patients younger than 40 years have a much higher risk of posttraumatic redislocation compared with those over 40 years [5]. Other risk factors for recurrence include male gender, hyperlaxity, and, to a lesser extent, bony Bankart lesions, nerve palsies, and type of activity [5].

A concomitant fracture of the greater tuberosity (GT) is seen in approximately 20% (range: 15.5–25%) of patients presenting with anterior shoulder dislocation [6–8]. Inherent muscle tension of the attached rotator cuff (RC) displaces the fragment dorsally and cranially from the dislocated humeral head fragment [9]. The degree of fragment displacement after reduction has been suggested to be a prognostic factor regarding restitution of shoulder function. While nondisplaced and minimally displaced fractures can be treated conservatively, surgical fixation is indicated for 3–5 mm of displacement, depending on the patient’s age and activity level [9–11].

We hypothesized that concomitant isolated fractures of the greater tuberosity are associated with low rates of recurrent dislocation but decreased range of motion (ROM) after primary traumatic anterior shoulder instability, as repeatedly mentioned in the literature [7, 8, 12]. The purpose of this study was to retrospectively evaluate the clinical and radiological outcome of patients treated for anterior glenohumeral (GH) dislocation in combination with an isolated fracture of the GT who underwent either surgical or nonsurgical treatment.

## Patients and methods

The current investigation is a retrospective cohort study, approved by the institutional ethics committee (415-EP/73/501-2014).

### Study population

We conducted a retrospective review of the institutional shoulder database. We included all patients with (1) traumatic anterior shoulder dislocation in association with an (2) isolated fracture of the GT, and a (3) minimum follow-up (FU) of 2 years. Exclusion criteria were: (1) any other fracture of the proximal humerus or the glenoid, (2) previous surgical intervention to the affected shoulder, and (3) pre-existing neurological or muscular deficiency affecting the injured shoulder. From July 2007 to July 2013, a cohort of 71 consecutive shoulders in 69 patients were identified. Five patients sustained another subsequent fracture of the proximal humerus due to a later traumatic event and were excluded from clinical FU, leaving a study population of 66 consecutive shoulders in 64 patients. Of these, 39 were male patients (61%) and 25 female patients (39%). Reported causes of the trauma were: fall in a domestic setting (*n* = 25), winter sports accidents (*n* = 12), fall during leisure time activity without involvement of a vehicle (*n* = 9), bicycle accident (*n* = 9), motor vehicle accidents (*n* = 5), occupational accidents (*n* = 3), and epileptic seizure (*n* = 3).

In all, 53 patients (55 shoulders, 83%) were available for FU. Five patients (8%) died from an unrelated cause and six patients (9%) could not be contacted because current contact information was lacking. Mean patient age at the time of the initial trauma was 56.5 ± 14.4 years (range: 15–84 years). The mean FU was 59.0 ± 20.7 months (range: 25–96 months).

The study population was divided into two groups according to treatment approach: (1) nonsurgical group (37 cases, 67%) and (2) surgical group (18 cases, 33%; Table 1).

**Table 1:** Comparison between the conservative and surgical groups

	Conservative group (*n* = 48)			Surgical group (*n* = 18)			*p*
	Mean	SD	Range	Mean	SD	Range	
Age at follow-up	62.8 y	14.8 y	60 y (28–88)	52.4 y	16.7 y	55 y (23–78)	0.052
Age at trauma	58.0 y	14.9 y	61 y (23–84)	47.44 y	17.3 y	56 y (15–71)	0.050
Follow-up time	58.4 m	19.9 m	67 m (25–92)	60.7 m	23.2 m	70 m (27–97)	0.874

*SD* standard deviation, *trauma* age at injury, *y* years, *m* months

### Patient care

Our diagnostic and treatment protocol for patients with GH dislocation and concomitant GT fracture consisted of clinical examination followed by biplane radiographic imaging (true anteroposterior [a. p.] view and y‑view) to confirm the diagnosis of GH dislocation and determine position of the humeral head and the fractured greater tuberosity. One careful attempt of closed reduction was usually performed in the emergency room with adequate analgesia. If unsuccessful, the GH dislocation was reduced with the patient under general anesthesia and relaxation in the operating room to prevent the risk of iatrogenic humeral fractures [13, 14]. Clinical examination and radiographic imaging were repeated to identify neurological changes and confirm the reduction as well as fracture morphology of the greater tuberosity. In the case of inconclusive radiographs, an additional computed tomography (CT) scan with three-dimensional (3D) reconstruction to evaluate the fracture morphology and degree of GT displacement was obtained (42 patients).

Nerve injury became evident in seven patients during the initial examination. Of these patients, four were found to have axillary nerve palsy, two had radial nerve palsy with one affecting the brachial plexus. All of them fully recovered at a mean of 6 weeks after reduction.

Surgical treatment was indicated in (1) patients younger than 65 years of age and displacement greater than 3 mm and in (2) patients older than 65 years and displacement greater than 5 mm on initial CT, measuring the widest distance. Surgical treatment was furthermore indicated for patients with depression-type fractures and an irreducible fragment dislocation after reduction of the dislocated shoulder [10, 11].

Otherwise, nonsurgical treatment was advised, consisting of initial immobilization of the GH joint in internal rotation for 4 weeks with an arm sling and weekly clinical and radiological FU examination in our clinic to detect possible late displacement of the GT. This was followed by mobilization of the upper extremity under the guidance of a physical therapist starting with passive ROM and advancing at approximately 6 weeks to active ROM.

**Fig. 1:**
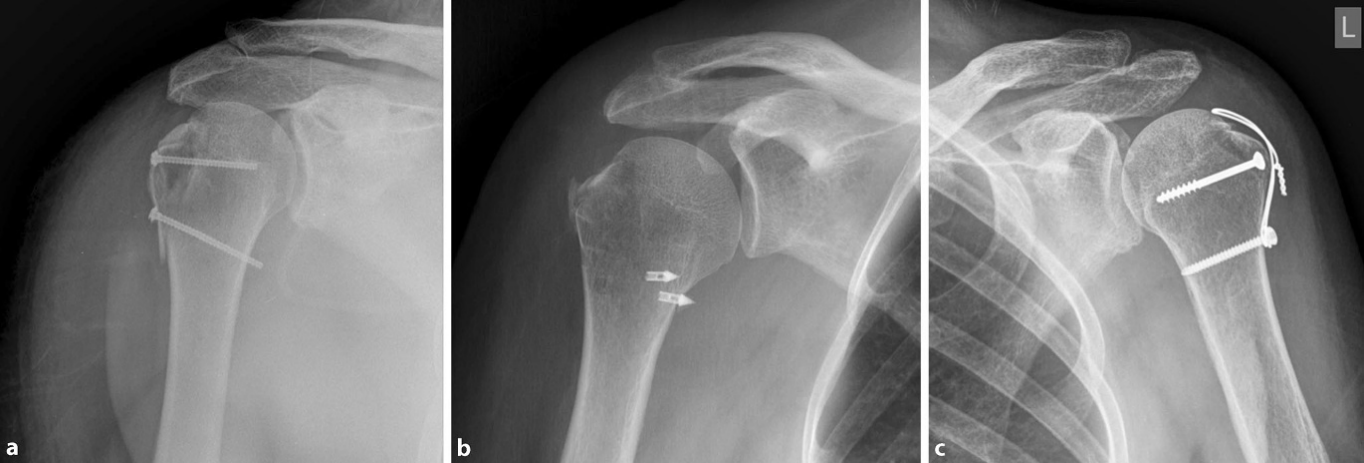
Surgical fixation techniques: **a** percutaneous reduction and fixation using 3‑mm cannulated self-tapping screws; **b** open reduction and fixation using sutures and suture anchors in a lateral single-row configuration; **c** screw fixation in combination with wire cerclages

Surgical treatment was performed either via percutaneous reduction of the fracture and fixation using cannulated self-tapping 3‑mm screws (7 cases; Fig. 1a) or open reduction and fixation with either cannulated self-tapping 3‑mm screws or suture anchors (6 cases; Fig. 1b) or screw fixation in combination with steel-wire cerclages (5 cases; Fig. 1c). All procedures were performed or supervised by a senior trauma surgeon. Postoperative care followed the conservative therapy protocol as described earlier.

### Clinical follow-up evaluation

After obtaining informed consent, the clinical FU was conducted in our outpatient clinic by the principal investigator and supervised by a resident. All patients filled out a questionnaire in order to complete patient history comprehensively. The clinical outcome was determined using the subjective shoulder value (SSV; [15]), Western Ontario Shoulder Instability Index (WOSI; [16]), the Rowe scoring system (Rowe 1988; [17]), and the Constant and Murley score (CS; [18]). With regard to the heterogeneity of our study population, the “age- and gender-adjusted CS” was used [19]. The shoulder-related level of exertion in terms of work and sports was measured with the shoulder activity level (SAL; [20]). Current pain intensity was determined using a 10-part visual analogue scale (VAS 0–10).

Clinical examination included the assessment of the active ROM of both shoulders, which was measured with a goniometer including elevation, abduction (ABD), external rotation (ER), and internal rotation (IR) in 0° and 90° ABD position. Shoulder strength was assessed in 90° ABD position with an IDO isometer (IDO, Innovative Design Orthopaedics Limited, Redditch, Worcestershire, UK).

### Radiological evaluation

**Fig. 2:**
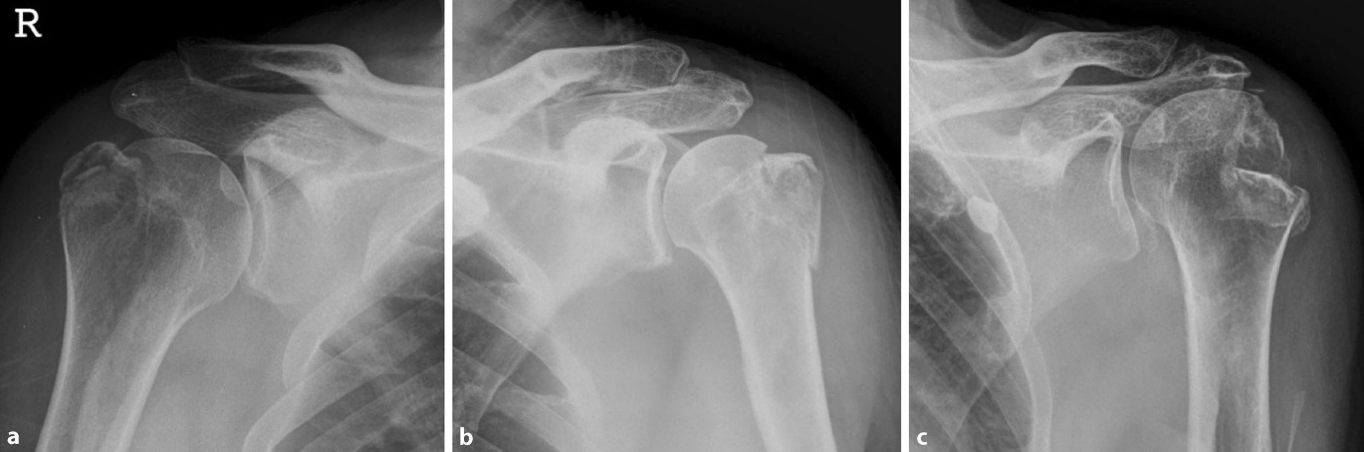
Morphological classification for greater tuberosity fractures: **a** avulsion type fracture; **b** split type fracture; **c** depression type fracture [21]

The type of fracture on imaging following reduction was categorized according to the fragment’s morphology into three groups applying a classification system for GT fractures [21]:

1. Avulsion fracture (Fig. 2a), characterized by small fragments and the presence of a horizontal fracture line caused by a shearing motion of the GT along the glenoid rim and tension of the muscles of the RC.
2. Split fracture (Fig. 2b), characterized by relatively large fragments and almost vertical fracture line from impaction of the GT on the anterior side of the glenoid.
3. Depression-type fracture (Fig. 2c), characterized by impaction of the fragment into the humeral head due to collision with the anterior glenoid. Depression-type fractures are distinct from Hill–Sachs lesions as the GT is entirely impacted into the humeral head, while Hill–Sachs lesions affect the posterolateral articular surface of the humeral head [22].

At the final FU, three views of radiographic imaging were obtained (true a. p. view, axillary view, and y‑view) to assess bony union, position, and displacement of the GT fragment and accurate articulation of the humeral head with the glenoid. Instability arthropathy was evaluated on true a. p. radiograph views of the shoulder according to the classification system of Samilson and Prieto [23]. FU images were compared with postreduction images to evaluate the development of instability arthropathy. We were able to radiographically examine 48 patients (73%), of whom 32 had undergone nonsurgical treatment and 16 cases had undergone surgical repair. All radiographic analyses were performed by a radiologist and a trauma surgeon.

### Statistical analysis

Statistical results were calculated with IMB® SPSS® Statistics Version 21. For all values, descriptive statistics were applied, using the mean, standard deviation (SD), and minimum and maximum values, while the Kolmogorov–Smirnov test was used to test for normal distribution. Comparison of the variables was made by using Student’s *t*-test for normally distributed data and the Mann–Whitney *U* test for nonnormally distributed data. The Kruskal–Wallis test was used to compare nonnormally distributed variables when there were more than two groups.

Statistical analysis was made with two-tailed *p *values and the alpha level was set at 0.05.

## Results

### Clinical evaluation

Clinical evaluation of 53 patients (55 shoulders) showed a mean CS of 75.1 ± 19.4 points (range: 25–100 points) and a mean age- and gender-adjusted CS of the affected shoulder of 94.2 ± 25.2% (range: 31.1–128.9%). The mean WOSI score was 373.2 ± 486.2 points (range: 0–2,078 points) and the mean Rowe score was 82.6 ± 19.5 points (range: 28–100 points). Pain intensity at FU averaged 1.6 ± 2.3 points (range: 0–8 points). The mean reported SSV for the affected shoulder was 78.9% ± 25.9% (10–100%). At FU the mean SAL reported by the study population was 1.6 ± 1 points (range: 0–4 points), whereat two patients reached an SAL of 4 points, 11 patients an SAL of 3 points, 14 an SAL of 2 points, 19 an SAL of 1 point, and nine an SAL of 0 points.

The ROM measured at FU is presented in Table 2. No statistically significant differences were detected between the nonsurgical and the surgical group in regard to ROM (elevation: *p* = 0.177; ABD: *p* = 0.178; ER neutral position: *p* = 0.703; ER 90° abduction: *p* = 0.651; IR 90° abduction: *p* = 0.307).

**Table 2:** Mean ROM and loss of ROM of the affected (index) shoulder vs. the unaffected (contralateral) side in conservative and surgical treatment groups

	Conservative (*n* = 37)				Surgical (*n* = 18)			
	Mean index	Contralateral	Mean loss	*p*	Mean index	Contralateral	Mean loss	*p*
*EL*	145°	155°	10°	0.024^*^	151°	160°	9°	0.058
*ABD*	144°	156°	12°	0.037^*^	150°	161°	11°	0.174
*ER 0°*	44°	53°	9°	0.037^*^	48°	59°	11°	0.027^*^
*ER 90°*	63°	73°	10°	0.004^*^	67°	78°	11°	0.013^*^
*IR 90°*	44°	54°	8°	0.131	51°	58°	7°	0.396

*ROM *range of motion, *EL* elevation, *ABD* abduction, *ER 0°* external rotation from neutral position, *ER 90°* external rotation from 90° abduction position, *IR 90°* internal rotation from 90° abduction position

*Statistically significant values (*p* < 0.05)

The results of the clinical outcome scores are outlined in Table 3.

**Table 3:** Comparison of clinical scores between surgical and conservative treatment subgroups

		Conservative (*n* = 37)			Surgical (*n* = 18)			
		Mean	SD	Range	Mean	SD	Range	*p*
*Score*	SSV	79.7%	27.7%	10–100%	80.94%	20.7%	30–100%	0.656
	WOSI	353.3	512.9	0–2078	356.78	391.3	10–1040	0.821
	ROWE	82.5	20.3	28–100	79.89	18.65	45–100	0.492
	CS	75.0	20.0	25–100	79.3	18.5	38–100	0.264
	CS_ag	94.7	26.1	31–125	94.4	21.1	45–129	0.815

*SSV* subjective shoulder value, *WOSI* Western Ontario Shoulder Instability Index, *ROWE* Rowe score, *CS* Constant–Murley score, *CS_ag* Constant score adjusted to age and gender, *SD* standard deviation

No statistically significant difference was detected between the three GT fragment type groups regarding the ROM (elevation: *p* = 0.203; ABD: *p* = 0.269; ER neutral position: *p* = 0.797; ER 90° abduction: *p* = 0.313; IR 90° abduction: *p* = 0.701) and clinical outcome scores (CS: *p* = 0.807; WOSI: *p* = 0.337; ROWE: *p* = 0.691; pain: *p* = 0.239; SSV: *p* = 0.467).

### Radiological evaluation

Review of the radiographs made after reduction of the GH dislocation demonstrated 29 (43.9%) avulsion type fractures, 26 (39.4%) split type fractures, and 11 (16.7%) impression type fractures according to the morphological properties [21] of the fractured tuberosity fragments (Table 4).

**Table 4:** Morphological classification of greater tuberosity fragments (according to Mutch et al. [21])

Fracture type	Cohort (*n* = 66)		Conservative (*n* = 48)		Surgical (*n* = 18)	
	*n*	%	*n*	%	*n*	%
*Avulsion*	29	43.9	23	47.9	6	33.3
*Split*	26	39.4	17	35.4	9	50
*Impression*	11	16.7	8	16.7	3	16.7

Of the initially 20 undisplaced fractures among the nonsurgical group, 19 remained in anatomical position and one underwent absorption. Of the initially four dorsally displaced tuberosities, three remained dorsally displaced, whereas one was found to be displaced dorsocranially at final FU. Of the initially two cranially displaced tuberosities, one was found in anatomical position at FU, whereas the other tuberosity was absorbed. Both dorsocranially displaced tuberosities showed no further evidence of subsequent displacement.

Review of postoperative radiographs in the surgical group showed three tuberosities with dorsal displacement, one tuberosity with dorsocranial displacement, and two impacted tuberosities, all of which remained in the same displaced position until final radiological FU. Of the ten anatomically reduced tuberosities, nine stayed in anatomical position at FU while one resorbed (Table 5).

**Table 5:** Greater tuberosity fragment position

Fragment position	Cohort (*n* = 48)		Conservative (*n* = 32)		Surgical (*n* = 16)		
	Follow-up	Posttreat.	Follow-up	Postred.	Follow-up	Postsurg.	Presurg.
*Anatomical*	29	30	20	20	9	10	0
*Absorption*	3	0	2	0	1	0	0
*Dorsal*	6	7	3	4	3	3	8
*Cranial*	0	2	0	2	0	0	2
*Dorsocranial*	4	3	3	2	1	1	4
*Impression*	6	6	4	4	2	2	2

*Posttreat.* after closed reduction or surgical procedures, *postred.* after closed reduction, *pre-/postsurg.* before/after surgery

Arthropathy was graded as “none” in 38 shoulders (79.2%), “mild” in eight shoulders (16.7%), and “moderate” in two shoulders (4.2%) of the conservative group on radiographs following reduction, while in the surgical group, arthropathy was graded “none” in all 18 shoulders. At FU, instability arthropathy among the conservative group was graded “none” in 18 shoulders (52.9%), “mild” in 11 shoulders (32.4%), and “moderate” in three shoulders (8.9%). Among the surgical group, nine shoulders (56.3%) were graded with “none” and seven shoulders (43.8%) with “mild” instability arthropathy at FU.

### Revisions

Revision surgery was necessary in five of 18 patients (27.8%); all of them were treated with cannulated self-tapping 3‑mm screws. One patient who was treated with percutaneous screws sustained screw breakage 13 days after surgery requiring revision surgery. Four other patients (22.2%) underwent removal of surgical implants owing to material migration, which was performed after an average time of 116.5 days (73–167 days). No patient of the nonsurgical group was scheduled for secondary surgical reduction and stabilization of the fragment after conservative therapy regime was started.

### Re-dislocation

Three cases (5.5%) of traumatic re-dislocation were reported among the study population, of which two cases were related to an epileptic seizure (bilateral re-dislocation in one patient). One case occurred during a traumatic skiing accident as the patient fell onto the previously injured shoulder. The formerly fractured GT remained stable during this second dislocation episode. All of the cases were observed in the conservative group.

## Discussion

The principal finding of the current study was that a concomitant isolated fracture of the greater tuberosity in cases with traumatic anterior shoulder dislocation was associated with a low recurrence rate but decreased ROM compared with the contralateral shoulder at mid-term FU.

In general, recurrent shoulder instability is the most common complication following primary shoulder dislocation with recurrence rates of up to 96% in adolescents [24]. Rates of recurrence are known to vary depending to a great degree upon the patient’s age, with recurrence rates of 54% in patients below 30 years and 12% for older patients [25]. The recurrence rate in this study cohort was much lower. A possible explanation might be the presence of the concomitant tuberosity fracture in all cases, which seems to reduce the risk for recurrence as previously described [10]. Other possible explanations are that the concomitant fracture of the greater tuberosity reduces the joint compression forces during the dislocation episode, which in return reduces the risk for damage to the anterior glenoid rim and anterior capsulolabral structures. Another explanation might be the observed loss of end-range of motion, which can also reduce the risk for instability [9, 10]. ROM, especially in external rotation and abduction, was significantly decreased on the affected side of our study patients compared with the nonaffected shoulder. No differences in ROM were seen in cases with surgical treatment compared with cases with conservative therapy when the aforementioned surgical indication criteria were applied. The mean loss of external rotation of approximately 10° in our study cohort is comparable to the outcomes after stabilization surgery for anterior shoulder instability [26].

The low recurrence rate of approximately 5.5% in the current study could partially also be explained by age-related factors. With increasing age, there is a higher risk of concomitant damage to bony structures such as the greater tuberosity during shoulder dislocation, which is most likely associated with reduced bone density at the proximal humerus. Therefore, in this study cohort primary shoulder dislocation occurred at an age of 40 years or older in about three out of four cases, which is much higher than the typically younger age at which primary traumatic dislocations occur [4]. Since the risk of recurrence decreases with increasing age at primary dislocation [5], the higher average age of the patients in this study can be considered as a confounder leading to a low recurrence rate.

Radiographic analysis at final FU revealed a low risk for secondary fragment displacement after both conservative and surgical treatment. In some cases, secondary fragment absorption was observed. Potential reasons for the absorption might be secondary dislocation with loss of strain on the tuberosity, lack of vascularity, or low-grade infection in the surgical cases.

Considering that the secondary displacement rate was low, the functional outcome was comparable, and the recurrence rate was low, conservative treatment in patients younger than 65 years and displacement less than 3 mm and in patients older than 65 years and displacement less than 5 mm seems to be justified, of course always taking into account the patient specific activity level, general health status, and severity of symptoms as well [27].

Evaluation of radiographs made after reduction of the GH dislocation with regard to morphological properties [21] of the fractured GT fragments demonstrated similar properties of avulsion type fragments (43.9%) and split type fragments (39.4%), whereas only a small proportion of impression type fractures were observed (16.7%). The proportions show almost the same distribution as those presented by Mutch et al. in their study in 2014 of 199 cases (avulsion type, 39%; split type, 41%; impression type, 20%; [21]). Statistical evaluation did not show any significant difference among these three subgroups regarding ROM and clinical outcome scores at FU.

### Limitations

The study has limitations typical of retrospective investigations. A control group of primary dislocations without concomitant fracture of the greater tuberosity was not available. Moreover, CT measurements of the distance between the tuberosity fragment and the intact proximal humerus were not available in all cases. Measuring the dislocation distance on radiographs might have limited reliability. Furthermore, no ultrasound examination or magnetic resonance imaging of the RC was made at FU, which could have offered more explanations for the loss of ROM.

Another limitation is the rather low number of surgical cases, which can result in a lack of statistical power when comparing results with the nonsurgical group.

## Practical conclusion

- Anterior GH dislocation with concomitant isolated fracture of the GT results in diminished joint mobility but low recurrence of instability.
- ROM in any direction was significantly decreased compared with the contralateral shoulder, regardless of whether the surgical or conservative treatment approach was followed.

## References

[1] Zacchilli MA, Owens BD (2010). Epidemiology of shoulder dislocations presenting to emergency departments in the United States. J. Bone Joint Surg. Am..

[2] Kroner K, Lind T, Jensen J, Krøner K, Lind T, Jensen J (1989). The epidemiology of shoulder dislocations. Arch Orthop Trauma Surg.

[3] Tas M, Canbora MK, Kose O, Egerci OF, Gem M (2013). Demographic and clinical characteristics of traumatic shoulder dislocations in an urban city of Turkey: a retrospective analysis of 208 cases. Acta Orthop Traumatol Turc.

[4] Rowe CR (1956). Prognosis in dislocations of the shoulder. J Bone Joint Surg Am.

[5] Olds M, Ellis R, Donaldson K, Parmar P, Kersten P (2015). Risk factors which predispose first-time traumatic anterior shoulder dislocations to recurrent instability in adults: a systematic review and meta-analysis. Br J Sports Med.

[6] Mattyasovszky SG, Burkhart KJ, Ahlers C (2011). Isolated fractures of the greater tuberosity of the proximal humerus: a long-term retrospective study of 30 patients. Acta Orthop.

[7] Kralinger FS, Golser K, Wischatta R, Wambacher M, Sperner G (2002). Predicting recurrence after primary anterior shoulder dislocation. Am J Sports Med.

[8] McLaughlin HL, MacLellan DI (1967). Recurrent anterior dislocation of the shoulder. II. A comparative study. J Trauma.

[9] Green A, Izzi J (2003). Isolated fractures of the greater tuberosity of the proximal humerus. J. Shoulder Elbow Surg..

[10] Bono CM, Renard R, Levine RG, Levy AS (2001). Effect of displacement of fractures of the greater tuberosity on the mechanics of the shoulder. J Bone Joint Surg Br.

[11] Park TS, Choi IY, Kim YH, Park MR, Shon JH, Kim SI (1997). A new suggestion for the treatment of minimally displaced fractures of the greater tuberosity of the proximal humerus. Bull Hosp Jt Dis.

[12] Hermodsson I (1934). Roentgenologischen Studien uber die traumatischen und habituellen schulterverrenkungen nach vorn und nach unten. Acta Radiol.

[13] Atoun E, Narvani A, Even T (2013). Management of first-time dislocations of the shoulder in patients older than 40 years: the prevalence of iatrogenic fracture. J Orthop Trauma.

[14] Demirhan M, Akpinar S, Atalar A, Akman S, Akalin Y (1998). Primary replacement of the humeral head in iatrogenically displaced fracture-dislocations of the shoulder: a report about six cases. Injury.

[15] Gilbart MK, Gerber C (2007). Comparison of the subjective shoulder value and the Constant score. J. Shoulder Elbow Surg..

[16] Kirkley S, Alvarez CAG (1998). The development and evaluation of a disease-specific quality of life measurement tool for rotator cuff disease: the western ontario rotator cuff index. J. Am. Acad. Orthop. Surg..

[17] Rowe CR, Rowe C (1988). Evaluation of the shoulder. The shoulder.

[18] Constant CR, Murley AH (1987). A clinical method of functional assessment of the shoulder. Clin Orthop Relat Res.

[19] Constant CR, Gerber C, Emery RJH, Søjbjerg JO, Gohlke F, Boileau P (2008). A review of the Constant score: modifications and guidelines for its use. J. Shoulder Elbow Surg..

[20] Moroder P, Odorizzi M, Pizzinini S, Demetz E, Resch H, Moroder P (2015). Open Bankart repair for the treatment of anterior shoulder instability without substantial osseous Glenoid defects. J. Bone Joint Surg. Am..

[21] Mutch J, Laflamme GY, Hagemeister N, Cikes A, Rouleau DM (2014). A new morphological classification for greater tuberosity fractures of the proximal humerus: validation and clinical implications. Bone Joint J..

[22] Hill HA, Sachs MD (1940). The grooved defect of the humeral head. Radiology.

[23] Samilson R, Prieto V (1983). Dislocation arthropathy of the shoulder. J. Bone Joint Surg. Am..

[24] Deitch J, Mehlman CT, Foad SL, Obbehat A, Mallory M (2003). Traumatic anterior shoulder dislocation in adolescents. Am J Sports Med.

[25] Vermeiren J, Handelberg F, Casteleyn PP, Opdecam P (1993). The rate of recurrence of traumatic anterior dislocation of the shoulder. Int Orthop.

[26] Di Silvestro MD, Lo IKY, Mohtadi N, Pletsch K, Boorman RS (2007). Patients undergoing stabilization surgery for recurrent, traumatic anterior shoulder instability commonly have restricted passive external rotation. J. Shoulder Elbow Surg..

[27] Moroder P, Scheibel M (2017). ABC classification of posterior shoulder instability. Obere Extremität.

